# Effect of short-term exposure to fluorescent red polymer microspheres on *Artemia franciscana* nauplii and juveniles

**DOI:** 10.1007/s11356-021-15992-y

**Published:** 2021-08-25

**Authors:** Diogo Peixoto, Amparo Torreblanca, Susana Pereira, Maria Natividade Vieira, Inmaculada Varó

**Affiliations:** 1 CIIMAR, Interdisciplinary Centre of Marine and Environmental Research, University of Porto, Av. General Norton de Matos s/n 4450-208, Matosinhos, Portugal; 2 Departament de Biología Funcional i Antropología Física, Universitat de València, Burjassot, Spain; 3 ICBAS-Instituto de Ciências Biomédicas Abel Salazar, Universidade do Porto, Porto, Portugal; 4 Department of Biology, Faculty of Sciences of University of Porto, Rua do Campo Alegre s/n, Edifício FC4 2.47, 4169-007 Porto, Portugal; 5 Instituto de Acuicultura Torre de la Sal (IATS-CSIC), Ribera de Cabanes, 12595 Castellón, Spain

**Keywords:** Microplastic, Brine shrimp, Biomarkers, Toxicity, Neurotoxicity, Oxidative stress

## Abstract

Microplastics (MPs) are ubiquitously present in the world’s seas with unknown potential toxic effects on aquatic ecosystems. The aim of this study was to evaluate biochemical responses caused by 1–5 μm diameter plastic fluorescent red polymer microspheres (FRM), under short-term exposure of nauplii and juveniles of *Artemia franciscana*, using a set of biomarkers involved in important physiological processes such as biotransformation, neuronal transmission and oxidative stress. Two FRM concentrations (0.4 and 1.6 mg mL^−1^) present in the water at ecologically relevant concentrations were used to study their toxicity. No significant differences were found in growth, survival and feeding behaviour of nauplii, after 2 days of exposure to both FRM concentrations. However, in juveniles, survival decreased after 5 days of exposure to FRM1.6; but no significant differences were found in either growth or feeding behaviour. It was observed that nauplii and juveniles, under short-term exposure, had the ability to ingest and egest FRM particles, although their accumulation was higher in nauplii than in juveniles, maybe related with the capacity of the latter to empty their gut content faster, in the presence of food. Regarding biomarkers responses in nauplii, all enzymatic activities increased significantly, after short-term exposure to the higher FRM concentration tested (FRM1.6), which could be related with detoxifying MPs-triggered oxidative stress. In juveniles, the inhibition of ChE and the decrease in the activity of antioxidant enzymes, after 5 days of exposure to FRM1.6, might indicate a neurotoxic effect and oxidative damage induced by FRM. This study provides further evidences that accumulation of MPs in the gut by nauplii and juveniles of *A. franciscana* can induce negative effects on important physiological processes with influence on their health, highlighting the general concern about the negative effects of MPs pollution on aquatic species, as well as the need to understand the mechanism of MPs toxicity and its possible impacts on environmental safety.

**Figure:**
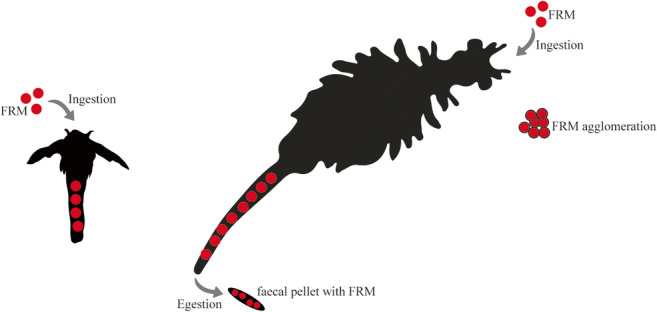
Graphical abstract

Graphical abstract

## Introduction

Plastics pollution and their pervasive fragments, such as microplastics (MPs), defined as plastic pieces that are <5 mm in size, are considered now more than ever an emerging environmental problem. MPs have the ability to adsorb persistent, bioaccumulative and toxic contaminants from the environment (Peixoto et al. 2019a), such as hazardous chemicals (Barboza et al. 2020; Kwon et al. 2017) including polycyclic aromatic hydrocarbons (PAHs) (Karami et al. 2017), metals (Fonte et al. 2016; Ma et al. 2016), toxins (van der Hal et al. 2020), pharmaceuticals (Fonte et al. 2016) and microorganisms (Foulon et al. 2016; Karami et al. 2017). Currently, the potential impact of MPs, as an environmental threat, has been gaining importance lately with an increasing number of studies demonstrating that they are a larger threat than previously thought. It has been shown that MPs can affect the marine food chains from the smallest planktivorous to the largest organisms and thus reaching high trophic levels including humans, with potential adverse effects even in their health, through the consumption of commercial products (Bouwmeester et al. 2015; Fossi et al. 2014; Peixoto et al. 2019b). Depending on their density and potential biofouling, MPs when present in water can be buoyant and occur in the first 0–3 m of water columns, being available to plankton and other animals (Kooi et al. 2017; Kooi et al. 2016). Zooplankton, as primary consumers in the food web, is a crucial food source for many secondary consumers; consequently, this represents a route whereby microplastic can enter the food web and transfer up the trophic levels. A number of toxicological studies carried out with several zooplanktonic species, under laboratory conditions, have confirmed the ingestion of MPs (Cole et al. 2015, 2013; Jeong et al. 2016; Lee et al. 2013; Peixoto et al. 2019a). In addition, natural ingestion of MPs by zooplankton has been observed (Sun et al. 2016). Ingestion of MPs by aquatic organisms can cause mortality but also induce alterations at physiological, biochemical and molecular levels. When ingested, MPs either on their own (Jeong et al. 2017) or due to their ability to adsorb persistent, bioaccumulative and toxic contaminants from the environment can generate reactive oxygen species (ROS), inducing mitochondrial dysfunction (Lee et al. 2016) and neurotoxicity (Barboza et al. 2018a; Miranda et al. 2019).

The brine shrimp *Artemia*, despite of occurring naturally in hypersaline environments, is commonly used as a suitable model organism in laboratory studies on the effects and trophic transfer of microplastics in marine environments (Batel et al. 2016; Bergami et al. 2017; Peixoto et al. 2019a; Wang et al. 2019). The ingestion of MPs has been shown by nauplii and juveniles of bisexual and parthenogenetic species of *Artemia*, after short- and long-term exposures, with no apparent impacts on growth and survival (Peixoto et al. 2019a; Wang et al. 2019). Additionally, Varó et al. (2019) reported survival impairment in *Artemia franciscana* nauplii after polystyrene nanoparticles (PS-NPs) short-term exposure, but not in the growth and feeding behaviour in juveniles after long-term exposure. Eom et al. (2020) reported that the exposure of *A. franciscana* early stages to MPs could be detrimental for population maintenance through inhibition of cholinergic system and the induction of acute cell stress, including oxidative stress.

Plastic fluorescent red polymer microspheres (FRM, 1–5 μm diameter), are commercially available and were used as representative of MPs by their diameter and easy quantification in test medium and detection inside of the individuals of *A. franciscana.* Also, these MPs have application on biotechnology, medical and scientific research, being a suitable model of primary MPs, used in cosmetics and personal care products. In fact, the effects of FRM in several model organism reveal to have negative impacts, such as causing neurotoxicity, oxidative stress, damage, changes in the activities of energy-related enzymes and significant reduction of the swimming velocity and resistance time of *Dicentrarchus labrax* juveniles (Barboza et al. 2018a, 2018b), neurotoxicity and oxidative stress in *Pomatoschistus microps* juveniles (Fonte et al. 2016) and in freshwater bivalve *Corbicula fluminea* (Guilhermino et al. 2018).

In our previous study, a decrease in reproductive success (total offspring) of *A. franciscana* individuals was observed, after long-term exposure to ecologically relevant concentrations of FRM, which may lead to a reduction in population’s size (Peixoto et al. 2019a). However, ingestion of FRM may also cause sublethal responses, after short-term exposure, at different biological levels, which may differ depending on the developmental stages of *Artemia.* These sublethal responses can be detected and measured as early warnings before reproductive detrimental effects hamper population persistence. Recently, Wang et al. (2019) reported histological alterations of intestinal epithelial cells in *A. parthenogenetica* after short exposure (24h) to 10 μm polystyrene microspheres. Also, neurotoxicity and oxidative stress effects are described in *A. franciscana* after short- and long-term exposure to PS-NPs, as well as impairment expression of genes involved in cell protection, development and moulting (Bergami et al. 2017; Varó et al. 2019). Therefore, following our research on biological adverse effects of FRM, as a model of primary MPs, in brine shrimp *Artemia*, the aim of this study was to evaluate biochemical responses caused by 1–5 μm diameter FRM, under short-term exposure on *A. franciscana* nauplii and juveniles. A set of biomarkers involved in important physiological processes such as biotransformation, neuronal transmission and oxidative stress were investigated, together with growth, survival and feeding behaviour. FRM particles were selected given their widespread use and application (e.g. biotechnology, medical and scientific research), being a suitable model of primary MPs, used in cosmetics and personal care products (Martins and Guilhermino 2018).

Outcomes generated from this study are expected to provide a better understanding of the impact of MPs, at environmentally relevant aquatic concentrations, using brine shrimp *Artemia* as a suitable model organism.

## Materials and methods

### *Artemia* hatching and culture conditions

Newly hatched nauplii of *A. franciscana* were obtained from commercially dried cysts (INVE Company, Belgium). *Artemia* dried cysts were hatched in filtered (1-μm polypropylene filter/Atlas Filtri) natural seawater (NSW, 37 g L^−1^) at 28 °C under conditions of continuous illumination and aeration, following the general procedure described in Varó et al. (2015). After 20 h, newly hatched nauplii (Instar I) were collected and separated from their shells and unhatched cysts by siphoning in a 150-μm mesh, washed thoroughly with NSW and transferred to clean filtered (0.45-μm Whatman WCN/filters) NSW. To obtain instar II–III nauplii for short-term FMR exposure tests, part of the new newly hatched nauplii was transferred to glass flasks (150 mL) filled with filtered (0.45 μm) NSW at a density of 5 nauplii mL^−1^ and acclimated during 24 h, in a thermostatic chamber (25 ± 0.5 °C and 16:8 h light/dark photoperiod), with moderate aeration without being fed. The remaining nauplii were reared in filtered NSW (1 μm) at the same density (5 nauplii mL^−1^) in 40-L cylindrical methacrylate containers and maintained at 25–27° C, with moderate aeration and natural autumn photoperiod until juvenile stage was reached, before used for short-term FRM exposure tests. The microalga *Tetraselmis suecica* (200,000 cell mL^−1^) was used as feed, freshly added to the *Artemia* culture, every 2 days to maintain cell density.

### Short-term FRM exposure tests

FRM (Cospheric LLC®, USA; lot number: 4-1006-1053) were used as a representative of 1–5 μm diameter MPs. These red opaque spheres (1.3 g.cm^−3^ density) are detected by florescence (575 nm EX/607 nmEX) allowing their easy quantification in test medium.

Two experimental concentration of FMR were tested, 0.4 mg L^−1^ (FRM0.4) and 1.6 mg L^−1^ (FRM1.6). FRM concentrations were selected based on our preliminary results on *A. franciscana* (Peixoto et al. 2019a). From the selected FRM concentrations, only 0.4 mg L^−1^ can be considered ecologically relevant in marine environments (Barboza et al. 2018a; Goldstein et al. 2012). FRM1.6 was chosen considering a scenario where MPs pollution steadily increases and particles become more available to organisms in aquatic environments. Experimental solutions of FRM were prepared in filtered (0.45 μm) NSW from a stock solution 160 mg FRM L^−1^ in Milli-Q water, being sonicated by ultrasonic cold water bath prior to dilution.

#### *Artemia* nauplii

Short-term exposure (2 days) test with *Artemia* nauplii was performed according to Varó et al. (2019) with some minor modifications. Briefly, nauplii 24 hours old (instar II–III) were separated and randomly transferred into to new glass flasks (5 nauplii mL^−1^) filled with 200 mL of filtered (0.45 μm) NSW containing the FRM concentrations chosen for testing (FRM0.4 and FRM1.6) and kept in the thermostatic chamber under the same conditions describe above (25 °C, 16:8 h photoperiod and moderate aeration), without being fed, during 48h. Also, a control group with NSW (CRTL) was included. All test glass flasks were covered to prevent water evaporation. Each experimental FRM concentration and control was performed with six replicates, with 1000 individuals exposed per treatment. Survival was measured at the end of exposure assay (2 days). The growth (body length, mm) was determined on 30 individuals per treatment from photographs taken with a Leica MZ6 stereo microscope equipped with a Leica hz6 digital camera via the Leica Application Suite (LAS v4.12) software. Images were analysed using the open-source ImageJ software (version 1.52a, National Institute of Heath, USA).

#### *Artemia* juveniles

To assess the effect of short-term FRM exposure (5 days) on *Artemia* juveniles (≈12 days old), a total of 1200 individuals were randomly transferred from cultures to glass flasks filled with 200 mL of filtered (0.45 μm) NSW containing 0, 0.4 and 1.6 mg L^−1^ of FRM (CTRL, FRM0.4 and FRM1.6, respectively). Each treatment consisted of 3 replicates of 100 juveniles each, kept in the thermostatic chamber as describe above (25 °C, 16:8 h photoperiod and moderate aeration) and fed with the microalgae *T. suecica* (200,000 cell mL^−1^). All test glass flasks were also covered to prevent water evaporation. Complete medium renewal with FRM and survival assessment was performed every 2 days. Total body length was determined, at the beginning and at the end of exposures, on 30 individuals following the same procedure as for the nauplii.

### Ingestion of FRM and feeding behaviour

Ingestion of FRM was assessed in nauplii and juveniles at the end of exposure time. To determine the number of FRM ingested by nauplii and juveniles, the number of particles present in the medium was quantified in a Neubauer improved cell counting chamber, under a Leica DMLB (PL FLUOTAR 40×/0.70) fluorescence optical microscope (Peixoto et al. 2019a).

Feeding behaviour, after short-term exposure to FRM, was evaluated as filtration and ingestion rates in nauplii and juveniles following the procedure described in Varó et al. (2019). A total of 25 nauplii and 12 juveniles from each concentration were randomly selected and placed in 12-well plates, filled with 1 mL of microalgae *T. suecica* at a density of 4×10^4^ cell mL^−1^ during 3 h. Blank samples in triplicate for each experimental group were prepared with the same volume of microalgal density without individuals. During the feeding experiment, the multiwell plates were placed in a thermostatic chamber (25 ± 0.5 °C) in total darkness. At the end of the feeding experiment (3 h), 1 mL from each well was collected, and the remaining algal cells were counted using a Neubauer chamber, and individuals discarded. The average of filtering (F) and ingestion rates (I) were calculated according to the formulae given by Gauld (1951), as described in Varó et al. (2019).

### Biomarkers assays

At the end of short-term FRM exposure tests, pools of ≈100 nauplii and 15 juveniles from each replicate were sampled, rinsed with distilled water and immediately stored at −80 °C for biomarkers analysis. Samples were processed according to previous studies (Varó et al. 2015, 2019). Briefly, samples were manually homogenized in 250 μL ice-cold buffer phosphate (100 mM pH 7.4, 150 mM KCl, 1 mM EDTA, 0.1% Triton X) using 250 μL of buffer *per* sample and centrifuged at 10000g at 4 °C for 15 min, and the resulting supernatants were stored in aliquots at −80 °C until enzyme analyses.

Cholinesterase activity (ChE) activity was measured using the modified Ellman method using acetylcholine (ATC) as substrate, and the absorbance was read at 415 nm during 15 min. Carboxylesterase activity (CbE) was determined according to Mastropaolo and Yourno (1981), using p-nitrophenyl acetate (pNPA) as substrate, and the absorbance was read at 405 nm. Glutathione-S-transferase activity (GST) was determined according to Habig et al. (1974) using 1-chloro-2,4-dinitrobenzene (CDNB) as substrate, and the absorbance was read at 340 nm during 5 min. Catalase activity (CAT) was determined by measuring the decrease of H_2_O_2_ concentration (Aebi 1974) using the procedure described in Varó et al. (2019), and the absorbance was read at 240 nm in UV microplate (Greiner UV-Star®) for 1 min. Glutathione reductase (GR) activity was determined measuring the decrease in absorbance at 340 nm for 3 min of oxidized glutathione (GSSG; 0.9 mM)) as substrate and nicotinamide adenine dinucleotide phosphate (NADPH; *ε* = 6.22 mmol^−1^ cm^−1^) as cofactor (Carlberg and Mannervik 1985), following the methodology described previously in Solé et al. (2015). Total protein content of samples was determined using Bradford Bio-Rad Protein assay, adapted to microplate and the absorbance was read at 595 nm.

All enzymatic activities were determined in triplicate at 25 °C in 96-wellplate using the kinetic mode on a TECAN Ultra Evolution microplate reader. The results are expressed as nmol of substrate hydrolysed per minute per milligram of protein (ChE, CbE, GR and GST) or μmol of substrate hydrolysed per minute per milligram of protein (CAT).

### Statistical analysis

Homogeneity and normality of variances of data were tested with Levene and Kolmogorov-Smirnov tests, respectively. Survival of juveniles after 5 days of exposure to different FRM concentrations was analysed using Kaplan-Meier curves (GraphPad 8), and log-rank (Mantel-Cox) test was used for pairwise comparisons of survival curves. The effect of short-term exposure to FRM on growth, microplastic ingestion, feeding behaviour and biomarker responses in nauplii and juveniles was assessed by one-way ANOVA, followed Student-Newman-Keuls post hoc test to check differences among experimental groups. Also, *t*-test was used to check differences in microplastic ingestion between stages. Statistical analyses were carried out using IBM SPSS v26.0 Statistics. All results are expressed as mean ± standard deviation, and the significance level was set at 95% (*p* ≤ 0.05).

## Results and discussion

### FRM effects on survival and growth

**Figure 1.**
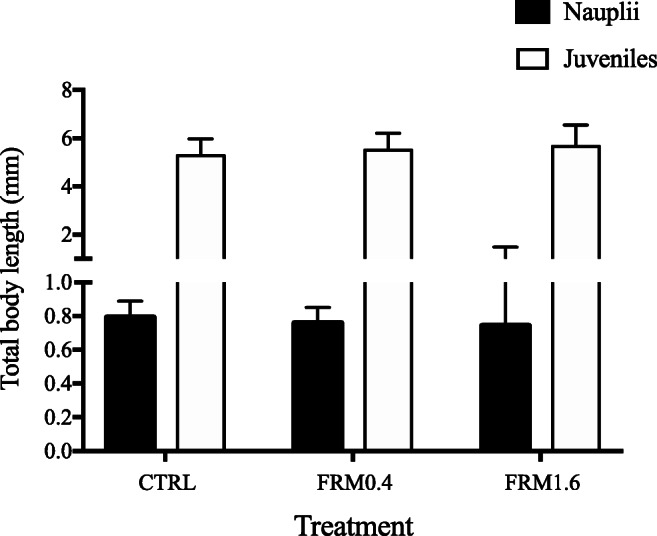
Growth (total body length, mm) of *A. franciscana* nauplii and juveniles, after 2 and 5 days of exposure, respectively, to different concentrations of plastic fluorescent red polymer microspheres (FRM): CTRL (0 mg L^−1^), FRM0.4 (0.4 mg L^−1^) and FRM1.6 (1.6 mg L^−1^).

The survival of nauplii was not affected after short-term exposure (2 days) to plastic fluorescent red polymer microspheres (FRM), with 90 % values in all experimental groups. Growth (total body length) was also unaffected by short-term exposure to FRM, with mean values ranging from of 0.79 mm for CTRL to 0.76 and 0.74 mm for FRM0.4 and FRM1.6 groups, respectively (Figure 1). Moreover, no visual changes in the development of nauplii were detected after 2 days of MPs exposure. Our survival and growth results are aligned with previous studies on early stages of different species of brine shrimp, after short-term exposure to different sizes and concentrations of microplastic. Also, the accumulation of microparticles has been confirmed inside the gut. Gambardella et al. (2017) reported that *A. franciscana* nauplii survival was not affected after 24-h exposure to 0.1-μm polystyrene beads, at any concentration tested (from 0.001 to 10 mg L^−1^). Similarly, Suman et al. (2020) also found that survival, growth and development of *A. franciscana* nauplii were not affected, after 2 days of exposure, to higher concentration (100 mg L^−1^) of 5 μm polystyrene particles. However, Kokalj et al. (2018) observed an effect on growth, but not on survival, using higher size microplastics (20–250 μm) and the same concentration. Likewise, for *A. parthenogenetica* nauplii, Wang et al. (2019) did not find effects on survival, growth or development, after 24-h exposure, to a wide range of 10 μm polystyrene concentration (0.55 to 550 mg L^−1^).

**Figure 2.**
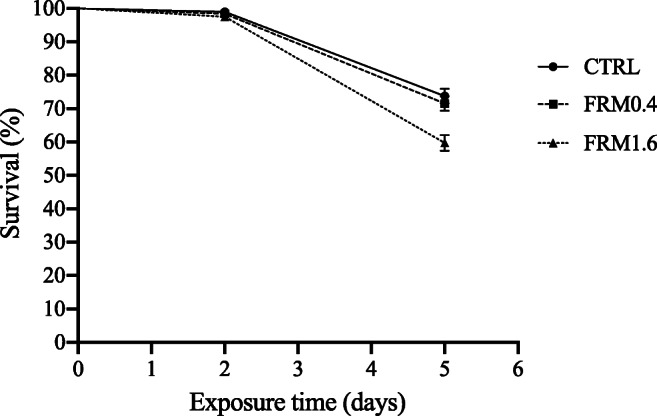
Survival of *A. franciscana* juveniles after 5 days of exposure to different concentrations of plastic fluorescent red polymer microspheres (FRM): CTRL (0 mg L^−1^), FRM0.4 (0.4 mg L^−1^) and FRM1.6 (1.6 mg L^−1^).

On the contrary, juvenile’s survival was significantly (log-rank, *p* ≤ 0.05) affected after 5 days of exposure (Figure 2), where survival of individual exposed at the highest FRM concentration tested (FRM1.6) was significantly lower (58.5%) compared to CTRL (73.6%) and FRM0.4 (71.4%) groups (Pairwise comparisons *p* ≤ 0.05). However, no effects of FRM on the total body length of juveniles or visible morphological changes were found, after 5 days of exposure (Figure 1). This is coincident with our previous results on *A. franciscana* growth after exposure to the same FRM concentrations, under long-term exposure (Peixoto et al. 2019a).

**Figure 3.**
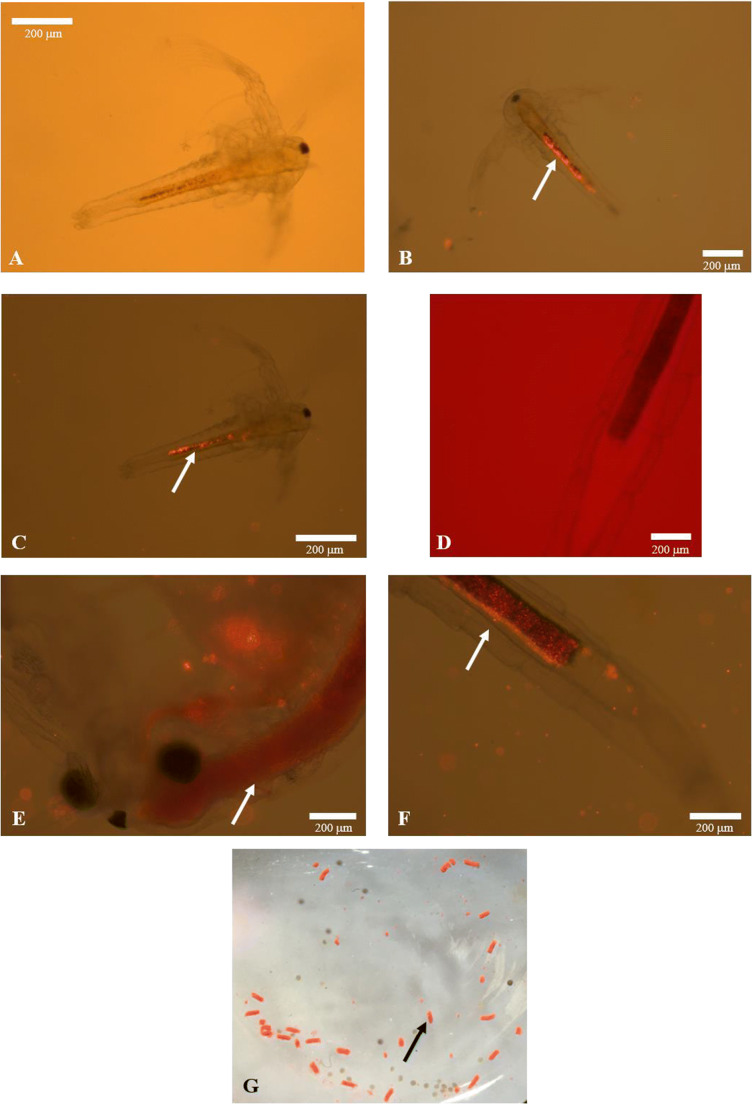
Plastic fluorescent red polymer microspheres (FRM, 1–5 μm diameter) ingested, egested and adhere (white arrows) to *Artemia franciscana* nauplii and juveniles and their aggregation to faecal pellets (black arrow), visualized using fluorescence microscopy. Digestive tract of nauplii after 2 days of exposure to different FRM concentrations: **A** CTRL (0 mg L^−1^), **B** FRM0.4 (0.4 mg L^–1^) and **C** FRM1.6 (1.6 mg L^−1^). Digestive tract of juvenile after 5 days of exposure to different FRM concentrations: **D** CTRL (0 mg L^−1^), **E** FRM0.4 (0.4 mg L^−1^) and **F** FRM1.6 (1.6 mg L^−1^). FRM aggregation into faecal pellets and sunk at the bottom of glass flaks (**G**).

In contrast with our previous study, where survival of juveniles was not affected after long-term exposure to different FRM concentrations (Peixoto et al. 2019a), here the survival of juveniles decreased after short-term exposure to FRM1.6 (1.6 mg L^−1^). These results suggest that as rapidly growing larval organisms undergoing metabolic and morphological changes at early stages, sensitivity to microparticles exposure can change with brine shrimp development and time of exposure (Libralato et al. 2016; Mesarič et al. 2015). However, it may also be related to the observed presence of more particles attached to the appendages and body surface of juveniles, with increasing concentrations of FRM (Figure 3E–F), which may lead to a restriction in swimming and feeding, resulting in higher mortality. It should be kept in mind that the juvenile stage is characterized by the presences of 11 pairs of thoracopods, short antennas which have lost their long setae (Amat 1985); and that as filter feeders, they feed continuously while swimming by ingesting suitable size particles from the medium, through the telopodites of their thoracopods (Fernández 2001). It is important to note that despite the FRM aggregation into faecal pellets at the bottom of the glass flask (Figure 3G), medium renewal assured the availability of FRM microspheres for *Artemia* individuals directly from water, and that may be the cause of the higher mortality observed for juveniles at the FRM1.6 experimental group.

### FRM ingestion and feeding behaviour

**Figure 4.**
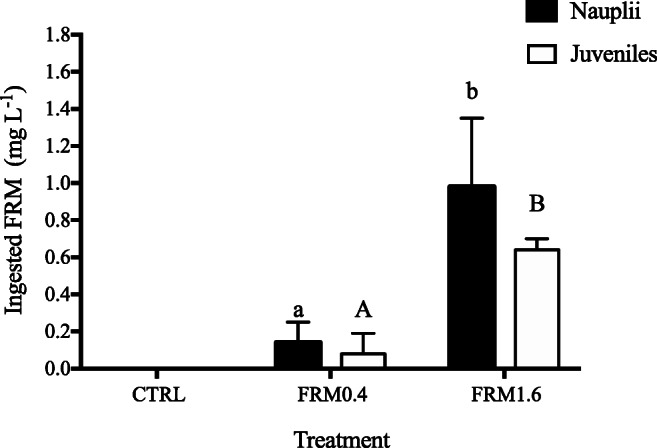
Concentration of ingested plastic fluorescent red polymer microspheres (FRM) (mg L^−1^) in *A. franciscana* nauplii and juveniles, after 2 and 5 days of exposure, respectively, to different concentrations: CTRL (0 mg L^−1^), FRM0.4 (0.4 mg L^−1^) and FRM1.6 (1.6 mg L^−1^). *N* = 4 replicates per treatment. Different letters show significant differences among treatments for nauplii and juveniles (ANOVA and Student-Newman-Keuls post hoc test *p* ≤ 0.05).

FRM ingestion by *A. franciscana* nauplii and juveniles as well as their adherence to the body surface was confirmed by fluorescence microscopy (Figure 3). FRM particles were found in the digestive tract of nauplii and juveniles after short-term exposure (Figure 3 B, C, and F), as well as in the gastric caeca of juveniles (Figure 3E). The number of ingested FRM increased significantly (*p* ≤ 0.05) with increasing concentration in both stages (Figure 4). No differences in the number of particles ingested between nauplii and juveniles were observed for the same FRM concentration. However, the accumulation of FRM particles was higher in nauplii than in juveniles.

Previous studies have confirmed the presence of microplastics inside of the gut and their adherence on the organisms’ surface at different stages in brine shrimp *Artemia*, after short- and long-term exposure (Kim et al. 2021; Kokalj et al. 2018; Peixoto et al. 2019a; Suman et al. 2020). It is known that *Artemia*, as non-selective filter-feeder, has the ability to ingest particles smaller than 50 μm readily. However, Fernández (2001) showed that the food for *Artemia* would range between 6.8 and 27.7 μm, with an optimum about 16 μm. Indeed, it has been reported that *A. franciscana* can ingest indigestible sand particles ten times faster than algae of the same size, forming faecal pellets of undigested food that sink very quickly to the bottom (Evjemo and Olsen 1999; Reeve 1963a, 1963b). Micro- and nanoplastics are also known in literature for their ability to agglomerate in seawater and to adhere between them or to external appendages in filter feeding organisms (Cole et al. 2015, 2013), including brine shrimp *Artemia* (Mesarič et al. 2015; Peixoto et al. 2019a; Varó et al. 2019) (see Figure 2E–F). In this study, as in our previous research (Peixoto et al. 2019a), the ingestion of FRM (1–5 μm diameter) in nauplii and juveniles in short-term exposures has been shown. These results are supported by previous MPs exposure studies in brine shrimp *Artemia* (Ates et al. 2013a, 2013b; Bergami et al. 2017; Gambardella et al. 2014; Kokalj et al. 2018; Mesarič et al. 2015; Peixoto et al. 2019a; Suman et al. 2020; Varó et al. 2019; Wang et al. 2019) and in other zooplanktonic species (Cole et al. 2015; Cole et al. 2013; Coppock et al. 2019; Jeong et al. 2017). Also, ingested FRM beads are accumulated by both nauplii and juveniles and excreted as faecal pellets (Eom et al. 2020; Peixoto et al. 2019a; Suman et al. 2020). The higher accumulation of FRM particles observed in nauplii is in line with the results found by Suman et al. (2020), who describe higher accumulation of polystyrene microplastics in naupliar stages compared to adults, after chronic exposure (14 days). The differences found in the accumulation of FRM particles between nauplii and juveniles could be related to the absence of food during nauplii exposure to FRM. In fact, the gut clearing time in *Artemia*, as continuous filter feeders, is reduced at low food concentration (Evjemo and Olsen 1999), or in its absence, irrespective of size (Nimura 1989), and therefore could be favouring the accumulation of FRM particles inside of the gut of nauplii.

Regarding feeding behaviour, ingestion and filtration rates of nauplii and juveniles were not affected, after 2 and 5 days of exposure, respectively, to different FRM concentrations (Table 1). These results were similar to those achieved by Varó et al. (2019) after a short- and long-term exposure of *A. franciscana* individuals to different concentrations of nanoparticles (0.1 and 1 μg mL^−1^ of PS-NH_2_). An explanation for the lack of effects on feeding behaviour rates (cleaning phase) in nauplii and juveniles might be related with the capacity of brine shrimp *Artemia* to empty its gut content, egesting FMR particles in the presence of food, as reflected by the presence of FRM particles in faecal pellets or sunk at the bottom of glass flaks (see Figure 3G). Although in this study the egestion time of MPs was not determined, it has been shown that intestinal contents in *A. franciscana* are completely replaced in about 30 min, regardless of size, in the presence of food (Nimura 1989; Evjemo and Olsen 1999).

**Table 1.** Filtration and ingestion rates of *A. franciscana* nauplii and juveniles, after 2 and 5 days of exposure, respectively, to different concentrations of plastic fluorescent red polymer microspheres (FRM): CTRL (0 mg L^−1^) FRM0.4 (0.4 mg L^−1^) and FRM1.6 (1.6 mg L^−1^).

		**CTRL**	**FRM0.4**	**FRM1.6**	***p*-value**
**Nauplii**	Individual filtration rate (mL/ind/h)	182.597 ± 22.012	166.943 ± 21.394	164.988 ± 26.839	0.17
	Individual ingestion rate (cel/ind/h)	9.548×10^6^ ± 5.911×10^5^	9.477×10^6^ ± 5.686×10^5^	9.490×10^6^ ± 1.016×10^5^	0.24
**Juvenile**	Individual filtration rate (mL/ind/h)	562.333 ± 108.080	595.750 ± 104.803	624.500 ± 22.752	0.65
	Individual ingestion rate (cel/ind/h)	4.708×10^7^ ± 8.960×10^5^	4.699×10^7^ ± 1.141×10^6^	4.799×10^7^ ± 1.148×10^5^	0.31

Values are expressed as means ± standard deviation (SD; *N* = 25 for nauplii *per* treatment and *N* = 12 for juvenile *per* treatment). *p*-values from one-way ANOVA (*p* > 0.05).

### Biomarkers

**Figure 5.**
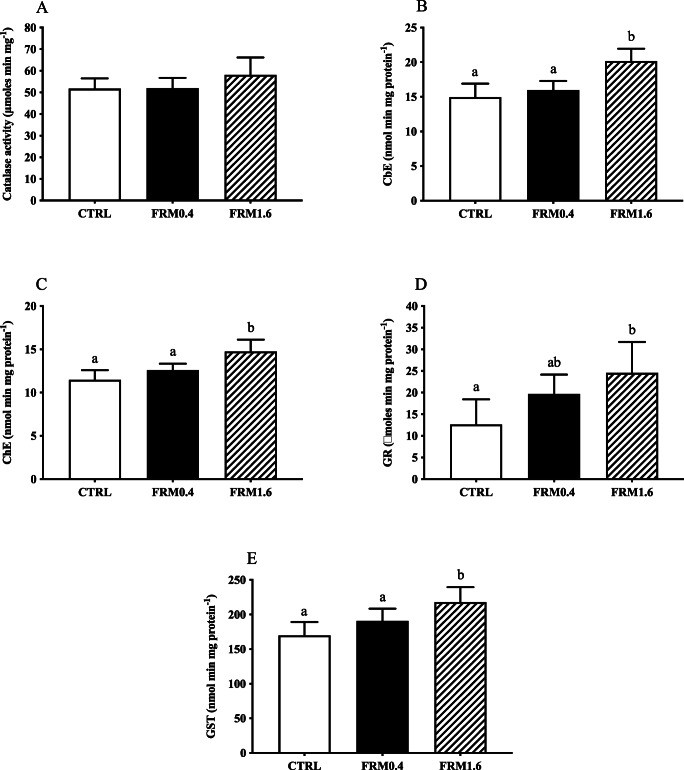
Biomarker responses (catalase (CAT), cholinesterase (ChE), carboxylesterase (CbE), glutathione-S-transferase (GST) and glutathione reductase (GR)) of *A. franciscana* nauplii, after short-term exposure to different FRM concentrations: CTRL (0 mg L^−1^) FRM0.4 (0.4 mg L^−1^) and FRM1.6 (1.6 mg L^−1^). Values are expressed as means ± standard deviation (SD). *N* = 6 pools of 100 individuals. Different letters denote significant differences among treatments (ANOVA and Newman-Keuls post hoc test, *p* ≤ 0.05).

**Figure 6.**
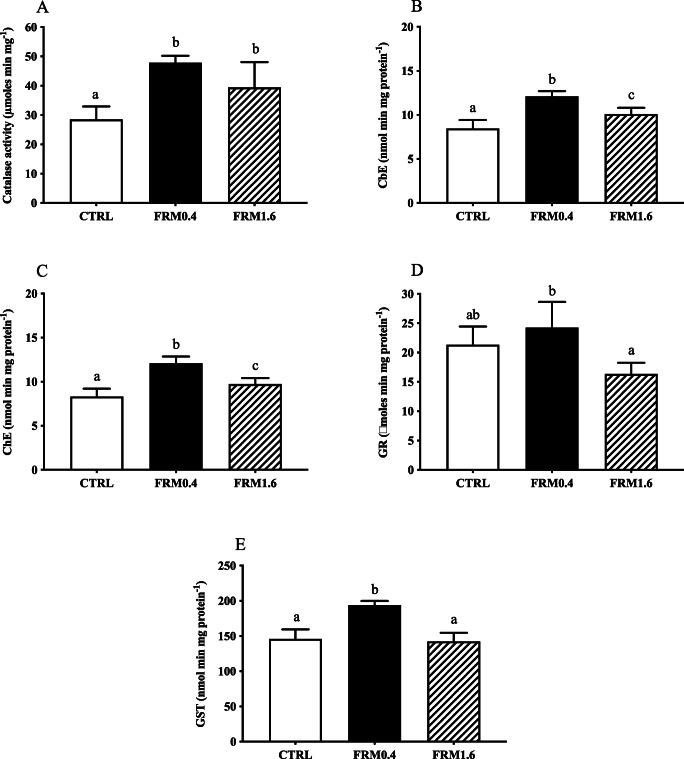
Biomarker responses (catalase (CAT), cholinesterase (ChE), carboxylesterase (CbE), glutathione-S-transferase (GST) and glutathione reductase (GR)) of *A. franciscana* juveniles, after short-term exposure to different FRM concentrations: CTRL (0 mg L^−*1*^), FRM0.4 (0.4 mg L^−*1*^) and FRM1.6 (1.6 mg L^−*1*^). Values are expressed as means ± standard deviation (SD). *N* = 4 pools of 15 individuals. Different letters denote significant differences among experimental treatments (ANOVA and Newman-Keuls post hoc test, *p* ≤ 0.05).

Biomarker responses of nauplii and juveniles after short-term exposure to FRM0.4 and FRM1.6 are presented in Figures 5 and 6, respectively. No concentration-dependent changes were observed in the enzyme activities determined at both stages. In nauplii, CAT activity was not affected by FRM after 2 days of exposure (Figure 5A). However, CbE, ChE, GR and GST activities increased significantly with respect to CRTL group (*p* ≤ 0.05) only in nauplii exposed to FRM1.6 (Figure 5 B, C, D and E).

For juveniles, all activities increased significantly (*p* ≤ 0.05) with respect to CTRL after 5 days of exposure to FRM0.4, followed by a decrease in the groups exposed to FRM1.6, which was significant respect to CTRL only for ChE and CbE activities. The exception was CAT activity, which remained high in both FRM concentrations tested (Figure 6).

As mentioned above, FRM are ingested by *A. franciscana* nauplii and juvenile individuals, permeating the cell membranes, and increasing their bioavailability (Suman et al. 2020). Countless biological processes produce ROS, causing cell and tissue damage and inflammation. Oxidative stress is an important mechanism of biological response against MPs toxicity (Jeong et al. 2016; Sharifinia et al. 2020; Yu et al. 2018), produced by higher bioavailability, as suggested by several studies in aquatic invertebrates (Cole et al. 2020; Eom et al. 2020; Jeong and Choi 2019; Lee et al. 2016; Suman et al. 2020; Varó et al. 2019). Ingestion of MPs by brine shrimp *Artemia* has been shown to cause damage and deformation of intestinal epithelial cells when exposed to different forms and concentrations of polystyrene microplastics (Wang et al. 2019; Suman et al. 2020), as well as changes in the expression of genes involved in cell protection, immune responses, development and moulting (Bergami et al. 2017; Eom et al. 2020; Suman et al. 2020; Varó et al. 2019). According to Suman et al. (2020) and Wang et al. (2019), the damage of intestine in brine shrimps affects the digestive function, reduces the metabolism energy and nutrient absorption and then causes the overproduction of ROS that leads to oxidative stress, eventually initiating inflammation and apoptosis (Cheng et al. 2015).

Catalase (CAT) is an antioxidant enzyme involved in important defence mechanisms of living organisms against oxidative damage by removing the excess of H_2_O_2_, which is metabolized to molecular oxygen and water (Downs et al. 2001; Rodrigues et al. 2017). The oxidative damage is induced by ROS which decomposes H_2_O_2_ promoting the activity of antioxidant enzymes (Rodrigues et al. 2017). In previous studies, CAT activity has been reported to increase in brine shrimp *Artemia* after short-term exposure at high MPs concentrations (Gambrandella et al., 2017; Eom et al. 2020). In this study, differences in the response of antioxidant defences (CAT, GST and GR) to FRM exposure were found between nauplii and juveniles. CAT activity was not affected after 2 days of exposure to increasing FRM concentrations. However, the levels of CAT increased in juveniles after 5 days of exposure to both FRM concentrations, which might be indicative of increased cellular levels of H_2_O_2_, as response to increased cellular ROS (Suman et al. 2020). GR and GST activities in nauplii increased after exposure to higher FRM concentration (FRM1.6), while in juveniles, the activities increased at lower FRM concentration (FRM0.4), followed by a decrease in the activities at higher FRM concentration. GR enzyme is responsible for the reversion of the oxidized form of glutathione, leading to the formation of two molecules of glutathione which can perform again their natural detoxification role (Nunes et al. 2006). Also, GST enzyme plays an important role in the detoxification process of xenobiotics, as well as in the defence against oxidative damage. Our results suggest that the induction of an antioxidant defence system in nauplii after short-term exposure to higher FRM concentration (FRM1.6) could be associated with detoxifying MPs-triggered oxidative stress, as reported by Eom et al. (2020).

In relation to ChE and CbE enzymes, both activities increased in nauplii after short-term exposure to high FRM concentration (FRM1.6), with not extensive mortality, stating the high tolerance of *A. franciscana* nauplii to ChE inhibition by different types of xenobiotics (Varó et al., 2002) and also suggesting that increase in CbE activity after exposure at FRM1.6, along with increase with GST activity, could be related with MPs detoxification processes to prevent oxidative damage (Eom et al. 2020). However, in juveniles, both activities increased at FRM0.4 and decreased at FRM1.6. The inhibition of both ChE and CbE activities at high concentration of FRM, after short-term exposure (5 days), may indicate a neurotoxic effect and reduced capacity in detoxification of MPs, resulting in increased mortality of exposed individuals. However, ChE inhibition at the higher concentration tested does not seem to affect feeding behaviour when individuals are fed with fresh microalgae in microplastic-free medium (Varó et al. 2019), probably because particle egestion is more rapid and effective in the presence of food (Nimura 1989; Evjemo and Olsen 1999). Our results are in line with previous studies where exposure of MPs and NPs inhibit AChE activity causing oxidative damage and death in *A. franciscana* (Gambardella et al. 2017; Eom et al. 2020; Varó. et al. 2019; Wang et al. 2019). Recently, CbE inhibition in *A. franciscana* after short- and long-term exposure to polystyrene PS nano-spheres (PS-NH_2_) has been associated to alterations in development and moulting. Although CbE is well-known by its detoxification functions, and by its role in the catabolism of juvenile hormones in insects, being the cause-effect relation between high levels of juvenile hormones or alike with an abnormal development (Willis et al. 1982), no abnormal development was observed on nauplii and juveniles after short-term exposure FRM, as reported by (Varó et al. 2019) for PS-NH_2_ exposure.

Previous studies have reported that brine shrimp *Artemia* at different stages had a higher sensitivity to toxicants after chronic exposure (Manfra et al. 2015; Rotini et al. 2015; Varó et al. 2019), compared to acute exposure (Libralato et al. 2016; Mesarič et al. 2015). Our results show that all biomarkers analysed in both nauplii and juveniles were significantly affected after short-term exposure to both FRM concentrations tested, with the exception of CAT activity in nauplii (Gambardella et al. 2017; Eom et al. 2020), suggesting that differences in response may be associated with the stage of development and with the extended period of exposure of juveniles (5 days). Overall, our results show a neurotoxic effect and changes in the antioxidant defence system that could prevent oxidative damage produced by short-term FRM exposure in both nauplii and juveniles of *A. franciscana*, as previously reported for brine shrimp *Artemia* exposed to MPs (Gambardella et al. 2017; Eom et al. 2020). However, the effects of ecologically relevant concentrations of FRM at the biochemical level are probably easier to detect over longer exposure periods, which are the most likely natural exposure scenarios (Suman et al. 2020; Varó et al. 2019).

## Conclusion

According to our results (1–5 μm), FRM present in the water at ecologically relevant (0.4 mg mL^−1^) and an extreme concentration (1.6 mg mL^−1^) does not affect their growth and feeding behaviour (filtration and ingestion rates) of *Artemia* nauplii and juveniles. Nonetheless, survival was affected in juveniles by increasing FRM concentrations after 5 days of exposure. The lower accumulation of FRM particles found in juveniles compared to nauplii may be related with the capacity of brine shrimp *Artemia* to empty its gut content fast, egesting FMR particles in presence of food, as well as to a lower availability by agglomeration in seawater with increasing concentration and with the high adherence to the body surface. This study confirmed that accumulation of FRM particles in the gut induces neurotoxicity and oxidative stress in juveniles, and the different response in the enzyme’s activities may prevent oxidative damage and may be associated with development stage and exposure time. These findings highlight the general concern about the negative effects of MPs pollution on aquatic species, as well as the need for further research to understand the mechanisms of MPs toxicity and its potential impact on environmental safety.

## Data Availability

The datasets generated for this study are available on request to the corresponding author.
